# Non-interventional Study Evaluating the Mobilization of Stem Cells by Plerixafor Before Salvage Autologous Stem Cell Transplant in Relapsed Multiple Myeloma (IFM-2015-03)

**DOI:** 10.1007/s44228-023-00030-0

**Published:** 2023-02-12

**Authors:** Zoe van de Wyngaert, Florent Malard, Cyrille Hulin, Denis Caillot, Clara Mariette, Thierry Facon, Cyrille Touzeau, Aurore Perrot, Philippe Moreau, Benjamin Hebraud, Tarik Kanouni, Farhad Heshmati, Delphine Lebon, Mohamad Mohty, Christian Chabannon

**Affiliations:** 1 Service d’Hématologie Clinique et de Thérapie cellulaire, APHP, Hôpital Saint Antoine, Paris, France; 2 Sorbonne Université, INSERM, Centre de Recherche Saint-Antoine (CRSA), 75012 Paris, France; 3 Hématologie Clinique et Thérapie Cellulaire, CHU Bordeaux, Bordeaux, France; 4 Hématologie Clinique, CHU Dijon, Dijon, France; 5 Hématologie, CHRU, Hôpital A. Michallon, Grenoble, France; 6 Service des Maladies du Sang, CHRU, Hôpital Huriez, Lille, France; 7 Hématologie Clinique, CHU Hôtel Dieu, Nantes, France; 8 Hématologie et Médecine Interne, CHU de Nancy, Nancy, France; 9 Institut Universitaire du Cancer, Toulouse, France; 10 Hématologie Clinique, CHU Montpellier, Montpellier, France; 11 Unité de médecine transfusionnelle, Hôpital Cochin, Paris, France; 12 Service d’Hématologie Clinique, CHU Amiens, Amiens, France; 13 Centre de Thérapie Cellulaire, Institut Paoli Calmette, Marseille, France

**Keywords:** Plerixafor, Salvage autologous transplant, Relapsed multiple myeloma

## Abstract

**Introduction:**

Despite the implementation of new therapeutic agents, management of relapsed multiple myeloma (MM) remains a challenge. Salvage autologous hematopoietic cell transplant (AHCT) remains a valid therapeutic option for eligible patients who achieve prolonged response after a first AHCT. However, a second graft is not always available, and these patients may need a second mobilization.

**Patients and Methods:**

This prospective, non-interventional, multicenter study aimed to collect data on the feasibility of salvage AHCT using a plerixafor-based hematopoietic cell mobilization in relapsed MM, according to the plerixafor label in France. Adult patients with relapsed MM eligible for a second AHCT and mobilized using granulocyte- colony stimulating factor (G-CSF) and plerixafor were included.

**Results:**

Of the 23 patients, 17 achieved a successful hematopoietic cell mobilization and 13 were able to proceed to a second AHCT. Median age was 62.9 years (min–max 51–71). Ten patients (77%) were male. Eleven (85%) received AHCT as a third-line treatment or more. Median time between first and second AHCT was 5.4 years (range, 2.6–16.3). Among 18 evaluable patients, mobilization was successful for 17 (94%) of them [95% CI 84–100], with no reported side effects. Among the 13 patients who underwent salvage AHCT, the median time to engraftment was 14 days (min–max 11–29). One-year progression-free and overall survival were 88.9% [95% CI 43.3–98.4] and 100%, respectively.

**Conclusion:**

This study demonstrated that plerixafor allows safe and efficient mobilization in relapsed MM patients who are candidates for a salvage AHCT.

**Trial Registration:**

NCT02439476 Registered 8 May 2015, https://clinicaltrials.gov/ct2/show/NCT02439476.

## Introduction

Multiple myeloma (MM) is an incurable disease that is characterized by the accumulation of clonal plasma cells in the bone marrow. While MM patients with relapsed disease may achieve responses to subsequent antimyeloma therapies, the duration of response usually decreases with successive relapses until resistant disease develops. Until recently, the median survival following relapse after initial therapy was relatively short [1]. The approvals and introduction in daily practice of immunomodulatory agents (IMiDS), novel generation proteasome inhibitors (PI), and anti-CD38 monoclonal antibodies have provided effective therapeutic options, which give patients with relapsed or refractory MM the prospect of a prolongation of overall survival (OS) and progression-free survival (PFS) [2, 3].

Nevertheless, almost all patients ultimately relapse, and no plateau has yet been observed in the survival curves. At the time of disease recurrence, there is not a standard salvage approach but, instead, various therapeutic options are available, including novel agent-based therapy, administered for a fixed duration of time or until progression [4].

When a frozen graft is available, it is also possible to repeat high-dose therapy in eligible patients who previously responded to the frontline application of high-dose melphalan and autologous hematopoietic cell transplant (AHCT). Over time, several reports have demonstrated the feasibility of this salvage strategy. Two prospective studies assessing second AHCT efficacy and feasibility have been published, although data are difficult to extrapolate, given the rapid change in salvage regimens given before and after AHCT. The first showed a benefit in PFS and OS (median PFS of 19 months and OS of 67 months) for patients treated with salvage AHCT, although compared to a suboptimal regimen (weekly cyclophosphamide) [5]. The second study compared salvage AHCT and continuous treatment with lenalidomide and dexamethasone. PFS and OS were better for patients who received a second transplant (*p* = 0.0087 and *p* = 0.0057 respectively) [6]. Other data come from retrospective studies and are based on single-center experiences with small numbers of selected patients. In this setting, PFS has been shown to range from 8 to 22 months, and the treatment-related mortality (TRM) was acceptable, ranging from 0 to 22% [7], similar to the TRM observed after first AHCT. Various prognostic factors for prolonged PFS have been described, such as the duration of response to the first high-dose therapy, quality of response, or the number of lines of therapy prior to salvage AHCT [7].

However, a frozen stem cell graft is not always available, and recollection is sometimes necessary to perform a second AHCT. The optimal CD34^+^ stem cell yield should be over 5 × 10^6^ CD34^+^/kg and the minimal threshold of 2 × 10^6^ CD34^+^/kg is mandatory to achieve sufficient engraftment [8]. In heavily pretreated patients, mobilization with G-CSF (granulocyte-colony stimulating factor) alone, also known as steady state mobilization, or with G-CSF combined with cyclophosphamide, can fail to yield a sufficient amount of CD34^+^ stem cells [9, 10].

Plerixafor reversibly inhibits CXCR4 binding to stromal cell-derived factor-1-alpha (SDF1α) and is an effective agent to mobilize CD34^+^ cells into the peripheral blood. In normal volunteers, administration of plerixafor after four to five days of G-CSF stimulation resulted in a 3- to 3.5-fold increase in circulating CD34^+^ cells. Two phase-3 studies demonstrated that the combination of G-CSF and plerixafor is superior to G-CSF alone for mobilizing hematopoietic progenitor cells in front-line or as salvage therapy in patients with MM [11, 12].

With this background, this prospective, non-interventional, multicenter study aimed to collect data on the feasibility of salvage AHCT using a plerixafor-based stem cell mobilization in relapsed MM, according to the plerixafor label in France.

## Methods

### Patients

Patients were recruited from sites in the collaborative Intergroupe Françophone du Myélome (IFM) group in France. Patients aged ≥ 18 years, with relapsed MM, able to receive a second AHCT were eligible. Eligibility to autologous transplantation was based on local guidelines and standard practice. All patients mobilized using G-CSF + plerixafor according to the plerixafor label in France were included. Plerixafor was given sequentially after the administration of G-CSF, assuming that collection would not succeed after G-CSF alone in these patients (preemptive). Exclusion criteria were age > 75 years, contraindication to AHCT or to the use of plerixafor. This study was registered at ClinicalTrials.gov (NCT02439476). All patients signed a written agreement to participate in the study. This study was conducted according to the principles of the Declaration of Helsinki.

### Intervention

Apheresis was performed according to standard practice and local guidelines. Once the autologous hematopoietic cell product was available, patients were scheduled to undergo AHCT conditioned by high-dose melphalan according to standard practice in each center.

### Study Design

This was a non-interventional study, and care was performed as usual, according to each center’s practice. No additional interventions were requested for the study purpose. The date for each visit and any data generated were recorded on the appropriate electronic case report form (eCRF). The study consisted of 3 periods: an early screening period; a mobilization phase, where stem cell mobilization was performed according to standard practice at each participating center; a follow-up visit, at the time of high dose melphalan administration and performance of autologous graft infusion, and then for 12 months after salvage AHCT. Patients were followed according to each center’s practice.

Subjects were enrolled over a two-year period. However, because of decrease of interest in auto-transplant for myeloma, during the COVID-19 pandemic, the study had to be discontinued prematurely.

### Endpoints

The primary endpoint was the percentage of patients achieving at least 2 × 10^6^ CD34 + /kg recipient body weight in one apheresis session after plerixafor-based hematopoietic cell mobilization. Secondary outcomes included: engraftment after AHCT (neutrophil and platelet recovery), time to disease progression, duration of response, PFS, OS, and incidence of adverse events. Time to neutrophil recovery was defined as the first of 3 consecutive days in which the absolute neutrophil count (ANC) exceeded 0.5 × 10^9^/L, and to platelet recovery as the first of 3 days with 20 × 10^9^/L or higher without a need for platelet transfusion during a 5-day period. OS was defined as the time from AHCT to death, regardless of the cause. PFS was defined as survival with no evidence of relapse or progression.

## Results

In total, 23 patients were included in this observational study across six IFM centers. Unfortunately, because of the COVID-19 pandemic, some centers were unable to provide data for some patients. One patient had to be excluded because he did not fulfill the inclusion criteria.

Overall, data regarding the primary endpoint of the study were available for 18 patients, of which only one failed to collect at least 2 × 10^6^ CD34 + /kg peripheral blood cell stem cells in one apheresis session after stem cell mobilization. Thus, the success rate for the primary endpoint was 94%, [95% CI 84–100]. No significant side effects were reported during the study period in relation to hematopoietic cell mobilization.

Among the 17 patients who achieved a successful hematopoietic cell mobilization, 13 were able to proceed to a second AHCT. Patient and transplant characteristics for these 13 patients are summarized in Table 1. Median age was 62.9 years (min–max 51–71). Ten patients (77%) were male. Eleven (85%) received AHCT as a third-line treatment or more. Median time between first and second AHCT was 5.4 years (range, 2.6–16.3). Median time between peripheral stem cell mobilization and AHCT was 50 days (range, 35–82). Median follow-up was 12.6 [interquartile range (IQR) 3.3–21] months after AHCT. Among transplanted patients, median time to obtain an ANC over 0.5 × 10^9^/L, was 14 days (min–max 11–29). One-year OS was 100%. One-year PFS was 88.9% [95% CI 43.3–98.4]. At last follow-up, no patient had died from this procedure. Patients’ outcomes are summarized in Table 2.

**Table 1:** Patient and transplant characteristics (*n* = 13)

Characteristic	Value
Sample size, no	13
Patient age at AHCT2, years, median (range)	62.9 (51–71)
Patient gender (male), *n* (%)	10 (77)
Isotype of multiple myeloma at diagnosis, *n* (%)	
IgG IgA Light chain Kappa Lambda	9 (70) 2 (15) 2 (15) 6 (46) 7 (54)
ISS stage at diagnosis, *n* (%)	
I III Not evaluated Missing	1 (7.5) 2 (15) 1 (7.5) 9 (70)
Year of second transplant, median (min–max) [IQR]	2017 (2014–2019) [2016–2017]
Status at transplant, *n* (%)	
CR 2 Advanced Missing	1 (7.5) 11 (85) 1 (7.5)
Melphalan dose (mg/m2), median (min–max) [IQR]	200 mg/m^2^ (140–200) [140–200]
Time from previous transplant, median (min–max) [IQR]	
Days Years	1975 (947–5944) [1374–2211] 5.41 (2.59–16.28) [3.77–6]
Time between mobilization and HCT, days	
Median (min–max) [IQR] Missing	50 (35–82) [54–44] 8

*AHCT2* second autologous hematopoietic cell transplant, *ISS* International Staging System, *CR2* second complete response, *IQR* interquartile range, *min* minimum, *max* maximum

**Table 2:** Outcome after second salvage transplant

Characteristic	Value
Median follow-up (months), median (95% CI)	12.6 (3.3–21)
Time to ANC > 0.5 G/L (days), median (min–max) [IQR]	14 (11–29) [12–15.7]
Overall Survival at 1 year, *n* (%)	100%
Progression-free survival at 1 year, % (95% CI)	88.9% (43.3–98.4)
Relapse incidence at 1 year, % (95% CI)	11.1% (0.5–40.6)
Non-relapse mortality at 1 year, % (95% CI)	0%

*ANC* absolute neutrophil count

## Discussion

A salvage auto transplant is a valid treatment option for MM patients who previously responded to the frontline application of high-dose melphalan followed by AHCT. Before the wide use of lenalidomide maintenance after transplant, the recommendation was to consider high-dose therapy and AHCT if the initial remission duration after the first transplant was longer than 18 months [13]. More recent guidelines suggest that patients achieving a first response longer than 36 months can be eligible to receive a second AHCT [4]. However, indications mostly remain at the clinician’s judgement. These patients can achieve a meaningful PFS period. Thus, a salvage AHCT is a valid option in selected patients. Therefore, the possibility of mobilizing sufficient peripheral blood stem cells to perform such salvage autologous transplantation remains an important issue.

Despite its relatively small size, this study clearly demonstrates that plerixafor, a well-established hematopoietic cell mobilizing agent, safely allows achievement of a remarkable success rate of 94% in relapsed MM patients who are candidates for a salvage AHCT, when used according to the plerixafor label in France. Moreover, patients who proceed to this salvage second transplant have a 100% engraftment rate at a relatively quick median duration of 14 days after graft infusion, while enjoying a 0% rate of TRM. These results are robust and in line with the known safety and efficacy of plerixafor. Indeed, plerixafor is known to allow high hematopoietic cell yield and a successful collection rate, without significant toxicity [14].

Daratumumab and other anti-CD38 monoclonal antibodies are now widely used in relapsed MM patients. It has been shown that steady state mobilization of hematopoietic cells in patients treated with daratumumab is often poor. Our study results underline the efficacy of plerixafor in relapsed MM patients. In patients who are expected to be poor mobilizers, defined by a cut-off at 20 CD34^+^/μL before mobilization, preemptive use of plerixafor has shown better results compared with a second mobilization attempt [15, 16].

Our study was impacted by the COVID-19 pandemic as, during the first few months, it was judged preferable for many physicians to avoid intensive treatments such as AHCT, which are associated with prolonged hospitalizations and deeper immunosuppression [17]. At that time, many centers favored outpatient care whenever possible. Due to these exceptional circumstances, our study could not recruit the required number of patients, and some data are missing. Today, our better knowledge of SARS-CoV2 manifestations and patient management, along with access to vaccines, and preventive or curative treatments, has allowed us to get back to optimal management of hematology patients.

Overall, these results are robust and in line with the known safety and efficacy of plerixafor, providing a solid background for designing new interventional prospective trials aiming to refine the role of second salvage autologous transplantation in the rapidly-moving myeloma therapy landscape.

## Data Availability

Data are available from the corresponding author upon reasonable request and with permission of the study sponsor.
